# Anti-Müllerian Hormone and Inhibin-A, but not Inhibin-B or Insulin-Like Peptide-3, may be Used as Surrogates in the Diagnosis of Polycystic Ovary Syndrome in Adolescents: Preliminary Results

**DOI:** 10.4274/jcrpe.3253

**Published:** 2016-09-01

**Authors:** Aylin Yetim, Çağcıl Yetim, Firdevs Baş, Oğuz Bülent Erol, Gülnaz Çığ, Ahmet Uçar, Feyza Darendeliler

**Affiliations:** 1 İstanbul University İstanbul Faculty of Medicine, Department of Pediatrics, Division of Adolescent Medicine, İstanbul, Turkey; 2 Biruni University Biruni Faculty of Medicine, Department of Gynecology and Obstetrics, İstanbul, Turkey; 3 İstanbul University İstanbul Faculty of Medicine, Department of Pediatrics, Division of Pediatric Endocrinology and Diabetes, İstanbul, Turkey; 4 İstanbul University İstanbul Faculty of Medicine, Department of Radiology, İstanbul, Turkey; 5 İstanbul University Cerrahpaşa Faculty of Medicine, Department of Public Health, İstanbul, Turkey; 6 Şişli Etfal Training and Research Hospital, Clinic of Pediatrics, Division of Pediatric Endocrinology and Diabetes, İstanbul, Turkey

**Keywords:** adolescent, anti-Müllerian hormone, inhibin-A, inhibin-B, insulin-like peptide-3, Polycystic ovary syndrome

## Abstract

**Objective:**

Polycystic ovary syndrome (PCOS) is a common endocrine problem in adolescents with an increasing prevalence of 30%. Pursuing new biomarkers with high specificity and sensitivity in the diagnosis of PCOS in adolescents is currently an active area of research. We aimed to investigate the diagnostic value of anti-Müllerian hormone (AMH), insulin-like peptide-3 (INSL3), inhibin-A (INH-A), and inhibin-B (INH-B) in adolescents with PCOS and also to determine the association, if any, between these hormones and clinical/laboratory findings related with hyperandrogenism.

**Methods:**

The study group comprised 53 adolescent girls aged between 14.5 and 20 years who were admitted to our outpatient clinic with symptoms of hirsutism and/or irregular menses and diagnosed as having PCOS in accordance with the Rotterdam criteria. Twenty-six healthy peers, eumenorrheic for at least two years and body mass index-matched, constituted the controls. Fasting blood samples for hormones [luteinizing hormone (LH), follicle-stimulating hormone (FSH), dehydroepiandrosterone-sulfate (DHEAS), androstenedione (D4-A), total/free testosterone (T/fT), sex hormone binding globulin (SHBG), AMH, INSL3, INH-A, INH-B] were drawn after an overnight fast.

**Results:**

In the PCOS group, 83% of the subjects were oligomenorrheic/amenorrheic and 87% had hirsutism. The LH, LH/FSH ratio, total T, fT, free androgen-index (FAI), DHEAS levels were significantly higher (p=0.005, p=0.042, p=0.047, p<0.001, p=0.007, p=0.014, respectively) and SHBG was significantly lower (p=0.004) in PCOS patients as compared to the controls. Although the INSL-3 and INH-B levels showed no difference between the groups (p>0.05), AMH and INH-A levels were found to be significantly higher in the PCOS group compared to the controls (p<0.001, p<0.001, respectively). In multiple linear regression analysis, WC SDS (p=0.028), logD4-A (p=0.033), logSHBG (p=0.031), and total ovarian volume (p=0.045) had significant effects on AMH levels, and LH (p=0.003) on INH-A levels. In receiver-operating characteristic analysis, the cut-off values for AMH and INH-A were 6.1 ng/mL (sensitivity 81.1%) and 12.8 pg/mL (sensitivity 86.8%), respectively, to diagnose PCOS. When AMH and INH-A were used in combination, the sensitivity (96.2%) increased.

**Conclusion:**

INSL3 and INH-B were not found to have diagnostic value in adolescents with PCOS. On the other hand, it was shown that INH-A could be used as a new diagnostic biomarker in addition to AMH.

**WHAT IS ALREADY KNOWN ON THIS TOPIC?**

One of the important biomarkers used to confirm the diagnosis of polycystic ovary syndrome (PCOS) and to manage the treatment process in adults and adolescent patients is anti-Müllerian hormone (AMH). However, new biomarkers (inhibin-A, inhibin-B, and insulin-like peptide-3) that may be used in the diagnosis and follow-up have also been found and are in the phase of investigation.

**WHAT THIS STUDY ADDS?**

The levels of insulin-like peptide-3 and inhibin-B were not found to have diagnostic values in adolescents with PCOS; however, it was shown that inhibin-A could be used as a new biomarker in addition to AMH.

## INTRODUCTION

Polycystic ovary syndrome (PCOS) is one of the most common endocrine problems in women in their reproductive period (1). Although the prevalence of PCOS is 5-10% in adulthood, it may increase up to 30% in adolescence (2,3). It is thought that this reported increased prevalence in adolescents shows a variance depending on the criteria used for the diagnosis of PCOS and arises from the polycystic appearance of the ovaries which may be observed in adolescents or from menstrual irregularities which are expected in anovulatory cycles (2). It has been shown that this rate may even increase up to 50% in obese adolescents (4).

PCOS is a syndrome rather than a disease, and it has a wide variety of symptoms and clinical presentations. The classical findings include chronic anovulation, clinical or laboratory hyperandrogenism, and a typical polycystic appearance of the ovaries on ultrasonographic examination. According to the Rotterdam criteria specified in 2003, at least two of these criteria should be present to make a diagnosis of PCOS (5,6). However, it is known that the reference range of androgen levels in adolescents is different from those of adults, that the multicystic/polycystic appearance of the ovaries may be encountered in healthy girls during this period, that anovulatory cycles may be observed for a long time after menarche, and finally, that findings including acne are observed frequently in adolescent girls (7,8). Therefore, pursuing a biomarker with a high specificity and sensitivity in the diagnosis of PCOS in adolescents is currently an active area of research.

One of the important biomarkers used to confirm the diagnosis of PCOS and to manage the treatment process in adult and adolescent patients is the anti-Müllerian hormone (AMH). However, it is possible that other biomarkers, many of which are still in the phase of investigation, may be used in the diagnosis and follow-up of PCOS. One of these is insulin-like peptide-3 (INSL3) which is a member of the relaxin/insulin family. INSL3 is produced by testicular Leydig cells in men (9). In women, it is synthesized in the ovary, particularly by the theca interna cells of antral follicles as well as by the corpora lutea and ovarian stroma, and INSL3 levels seem to reflect gonadal function (10,11). INSL3 levels are similar in prepubertal and postpubertal periods, but a significant increase is observed after pubertal onset (12). Some studies show that INSL3 expression appears to change with follicle development; it is present at higher levels in small antral follicles and at lower levels as follicles become preovulatory, which suggests a correlation with follicular maturation (13,14). In contrast, Hagen et al (12) reported that INSL3 levels better reflect products from large follicles rather than products from small follicles. These authors also state that INSL3 is a specific marker for theca cells surrounding larger follicles. There are studies reporting that INSL3 is significantly increased in women with PCOS (15,16). INSL3 levels have also been investigated in healthy adolescents (12,17), but no study has investigated the diagnostic value of INSL3 in adolescent patients with PCOS to date.

Inhibin-A (INH-A) and inhibin-B (INH-B), which are the bioactive forms of inhibins in the α-subunit, are synthesized in the granulosa cells of the ovaries (18). It has been suggested that in normal women, the intercycle rise of follicle-stimulating hormone (FSH) is responsible for the increased secretion of INH-B from the small antral follicles in the early follicular phase, while the midcycle luteinizing hormone (LH) increase stimulates INH-A secretion from the pre-ovulatory follicle (19). In the light of this information, INH-B may be expected to increase in patients with PCOS who have large numbers of small antral follicles; INH-A levels increase as the follicle grows, and therefore they may be expected to be normal or decreased in PCOS patients. However, conflicting results have been reported in studies conducted on adult women. Some studies have shown that INH-A does not change in patients with PCOS (20,21), whereas others have shown that it increases (22,23,24) or decreases (25,26,27). There are also similar controversial results related with INH-B levels (20,21,27,28,29,30). No study to date has investigated these hormones only in the adolescent age group.

In this study, we aimed to investigate the diagnostic value of AMH, INSL3, INH-A, and INH-B in adolescents with PCOS and also to explore the association between these hormones and the clinical/laboratory findings related with hyperandrogenism.

## METHODS

We studied 53 adolescent girls aged between 14.5-20 years who were consecutively admitted to pediatric endocrine outpatient clinic of Istanbul Faculty of Medicine between August 2014 to August 2015, with symptoms of hirsutism and/or irregular menses, and diagnosed as having PCOS in accordance with the Rotterdam Criteria (5). Adolescents with chronic diseases, thyroid hormone dysfunction, congenital adrenal hyperplasia, tumors, genetic syndromes and other endocrine disorders, and those who used medications that might potentially influence the biomedical assessments were excluded from the study. Twenty-six healthy peers who were followed up in the well-child and adolescent health care unit and were eumenorrheic for at least two years constituted the control group. Menstrual cycles were defined as oligomenorrhea if the cycle intervals were longer than 45 days and amenorrhea if the cycle intervals were longer than 3 months. In addition, we tried to match the two groups in terms of body mass index (BMI). A detailed medical assessment and medical history were obtained from all subjects. Birth weight and length of the participants, family history of PCOS, mothers’ age at the time of her first menstruation, and family history for type 2 diabetes were also recorded. Written consent was obtained from all parents and participants. The study was approved by the Ethics Committee of İstanbul University Faculty of Medicine (no. 2421).

**Clinical Evaluation**

Weight and height were measured by the same physician (A.Y.) in all subjects using a wall-mounted calibrated Harpenden stadiometer (Holtain Ltd., Crymych, UK) and an electronic scale (sensitivity at 0.1 kg level). BMI was calculated using the following formula: BMI=weight (kg)/height (m^2^). Waist circumference (WC) was measured at the midpoint between the lower margin of the last palpable rib and the top of the iliac crest using a non-stretch tape. Hip circumference (HC) was measured around the widest portion of the buttocks with the tape parallel to the floor, and the waist to hip ratio (WHR) was evaluated. Standard deviation scores (SDS) of these measurements were calculated using national data (31,32,33). The Ferriman-Gallwey (FG) scoring method was used to define clinical hyperandrogenism (34).

### Laboratory Evaluation and Biochemical Assays

Fasting blood samples for glucose, insulin, LH, FSH, dehydroepiandrosterone sulfate (DHEAS), androstenedione (D4-A), total testosterone (T), free testosterone (fT), sex hormone-binding globulin (SHBG), AMH, INSL3, INH-A, and INH-B were drawn between 08:00-08:30 a.m., after an overnight fast. Basal 17-hydroxyprogesterone (17OHP) and cortisol levels were measured to exclude an adrenal enzyme defect; free thyroxine (T_4_) and thyroid-stimulating hormone (TSH) levels were measured to exclude a thyroid hormone defect, and prolactin level was measured to exclude intracranial pathologies. After separation, serum samples were frozen immediately and stored at -80 °C until they were assayed. The free androgen index (FAI) was calculated using T and SHBG values [FAI=100x(T/SHBG)].

Serum LH and FSH concentrations were measured using an immunochemiluminometric assay; DHEAS and T levels were measured using a radioimmune assay (RIA) (Diagnostic Products Corporation, Los Angeles, CA, USA). D4-A levels were determined using a solid-phase, competitive chemiluminescent enzyme immunoassay (Siemens Healthcare Diagnostics Technical Products, USA). 17OHP and serum cortisol levels were measured using RIA and DIA immunoassays (Beckmann Coulter Company, Marseille, France and S.A. Nivelles, Belgium, respectively). SHBG levels were estimated by IRMA (Roche Diagnostic, Rotkreuz, Switzerland). Free testosterone levels were measured using an RIA (Beckman Coulter Company, Prague, Czech Republic). Serum INH-A, INH-B, AMH (AL105-I), and INSL3 (SED873Hu) levels were determined by an enzyme-linked immunosorbent assay (ELISA) (Beckman Coulter Company, Webstar, USA, and USCN-life, Houston, USA). LH and FSH had an intra-assay coefficient of variation (CV) of 4.8-7.5% and interassay CV of 5.4-10.7%, respectively. DHEAS had an intra-assay CV of 4.5 and an interassay CV of 5.5%. Testosterone had an intra-assay CV of 4.5% and an interassay CV of 6.4%. For the D4-A assay, the intra-assay CV was 3.2-9.4% and interassay CV was 4.1-15.6%. For the cortisol assay, the intra-assay CV was 5.2% and interassay CV was 8.7%. The 17-OHP assay had intra-assay and interassay CVs of 5%. For the SHBG assay, the intra-assay CV was 2% and interassay CV was 8.3%. The INH-A assay had an intra-assay CV of 3.4-5.6% and an interassay CV of 5.5-6.7%, and the INH-B assay had an intra-assay CV of 2.9-4.5% and an interassay CV of 5.5-7.5%. Values for the AMH assay were 1.9-4.0% (intra-assay CV) and 4.5-6.0% (interassay CV). The INSL3 assay had intra-assay and inter-assay CVs of <10% and <12%, respectively.

### Pelvic Ultrasound

Ultrasound (US) examinations were performed on the same day as the hormonal and biochemical determinations. Transabdominal pelvic US scans were performed in the PCOS group prospectively by the same experienced pediatric radiologist (O.B.E.) who was blinded to the clinical and laboratory findings of the subjects. No US was done in the control group. US was performed using a conventional full bladder Logiq 6 US scanner (General Electric Co., Milwaukee, WI, USA) and a 5-MHz convex-array broad-band transducer or a 7.5-MHz linear-array small parts transducer, depending on age and size. The three dimensions of the uterus [total uterine length (UL), anteroposterior (AP), and transverse diameters of the corpus], endometrial thickness (ET), and the three dimensions of each ovary (longitudinal, transverse, and AP diameters) were measured. Uterine and ovarian volumes were calculated according to the formula for ellipsoid bodies: longitudinal diameter x AP diameter x transverse diameter x 0.52. An ovary was defined as polycystic if there were 12 follicles or more, each 2-9 mm in diameter (35).

### Statistical Analysis

SPSS version 15 (Chicago, IL, USA) was used for statistical analyses. Results are given as mean ± SD or median [minimum-maximum]. Normality was assessed using the Kolmogorov-Smirnov test. The normality of continuous variables was examined using three different methods including variability coefficient, Kolmogorov-Smirnov, and skewness-kurtosis values; if two of these three methods showed normal distribution, the distribution was considered compatible with normal distribution.

Parametric and nonparametric tests were used for inter-group comparisons. Skewed data (AMH, INH-A, INH-B, T, fT, SHBG, 17OHP, D4-A) were transformed to normal distributions by calculating normal logarithms and natural logarithms. Chi-square test was used for categorical variables, while student’s t-test and Pearson’s correlation analysis was applied for continuous variables in independent groups. The Mann-Whitney U test was used for continuous variables that did not show normal distribution. The linear regression model was applied using backward stepwise method in the patients with PCOS considering AMH, and INH-A as a dependent variable.

The candidate biomarkers that showed a significant difference in inter-group comparisons were examined using the receiver operating characteristics (ROC) curve analysis and their diagnostic values (sensitivity and specificity) were calculated. When the type-1 error level was below 5% in the evaluation of area under the curve (AUC), the diagnostic value of the test was considered statistically significant. The net sensitivity and specificity rates of the combined use of AMH and INH-A in the diagnosis of PCOS were calculated (36).

## RESULTS

Thirty-four percent of the patients with PCOS (n=18) were amenorrheic, 49% (n=26) were oligomenorrheic, and 17% (n=9) were eumenorrheic. Hirsutism was present in 87% of the patients with PCOS (n=46), acne was present in 43% (n=23), alopecia in 25% (n=13), and acanthosis nigricans in 49% (n=26). The distribution of subjects with obesity/overweight and normal body weight was similar in the PCOS group (57%) and control group (50%) (BMI-matched) (p>0.05).

Although the mean age of the PCOS group was slightly higher compared with the control group (p=0.001), the age at the time of menarche was similar in both groups (p=0.397). The birth weight SDS, birth length SDS, and BMI SDS values were similar in both groups (p>0.05). The WC SDS value was statistically significantly higher in the PCOS group (p<0.001). The anthropometric measurements of the patient and control groups are shown in Table 1.

The hormone levels of the PCOS and control groups are presented in Table 2. The LH, LH/FSH ratio, total T, fT, FAI, DHEAS levels were significantly higher in the PCOS group (p=0.005, p=0.042, p=0.047, p<0.001, p=0.007, p=0.014, respectively); SHBG was found to be significantly lower in the PCOS group (p=0.004). Although the INSL-3 and INH-B levels showed no difference between the groups (p>0.05), the AMH and INH-A levels were found to be significantly higher in the PCOS group compared with the control group (p<0.001, p<0.001, respectively).

**Correlation Analysis**

Anthropometric measurements: INSL3 level showed no significant correlation with the anthropometric measurements, whereas the AMH level had a positive correlation with WC SDS and WHR (r=0.305, p=0.008; r=0.240, p=0.038), the INH-B level demonstrated negative correlations with BMI SDS, WC SDS, WHR (r=-0.426, p=0.001; r=-0.377, p=0.001; r=-0.242, p=0.034, respectively), and the INH-A level had a positive correlation with WC SDS (r=0.285, p=0.013). FAI was found to positively correlate with the FG score, BMI SDS, WC SDS, and WHR (r=0.623, p<0.001; r=0.535, p<0.001; r=0.433, p<0.001; r=0.299, p=0.014, respectively).

Hormones: There was a negative correlation between the INSL3 level and INH-A (r=-0.296, p=0.009). AMH was found to significantly correlate with LH, DHEAS, fT, D4-A, and INH-A (r=0.255, p=0.032; r=0.288, p=0.014; r=0.572, p<0.001; r=0.415, p=0.004; r=0.385, p=0.001, respectively). The INH-A level significantly correlated with LH, LH/FSH ratio, SHBG, DHEAS, and cortisol levels in addition to AMH and INSL3 (r=0.313, p=0.008; r=0.350, p=0.003; r=-0.261, p=0.031; r=0.347, p=0.003; r=0.359, p=0.002, r=0.385, p=0.001; r=-0.296, p=0.009, respectively). The INH-B level was found to significantly correlate only with FSH (r=0.247, p=0.035).

Ultrasonographic findings: AMH was found to significantly correlate with left ovarian volume and total ovarian volume (r=0.438, p=0.002; r=0.346, p=0.019, respectively). INH-A significantly correlated with right ovarian volume and total ovarian volume (r=0.333, p=0.024; r=0.315, p=0.033, respectively).

**Regression Analysis**

In the PCOS group, multiple linear regression analysis was performed to explore the effect of WC SDS, FSH, logD4-A, logSHBG, logT, and total ovarian volume on the level of AMH. Factors that had an effect on the level of AMH included (adjusted R2=0.284) WC SDS (β=-0.058, p=0.028), logD4-A (β=0.664, p=0.033), logSHBG (β=0.012, p=0.031), and total ovarian volume (β=-0.495, p=0.045) (Table 3). Multiple linear regression analysis was also performed to explore the effect of logAMH, INSL3, LH, logSHBG, and DHEAS on the level of INH-A. The only factor that had an effect on the level of INH-A (adjusted R2=0.157) was LH (β=2.023, p=0.003) (Table 4).

**Receiver Operating Characteristics Analysis for Anti-Müllerian Hormone and Inbibin-A**

**Figure 1:**
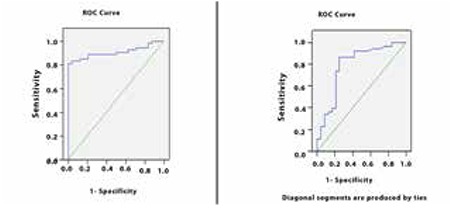
Receiver operating characteristics (ROC) curves of anti-Müllerian hormone (left) and inhibin-A (right). The sensitivity (y axis) is plotted against the specificity (x axis)

**Figure 2:**
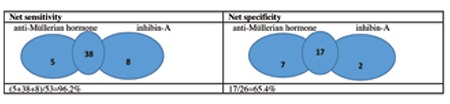
Net sensitivity and specificity when anti-Müllerian hormone and inhibin-A were used together

ROC curve analyses were performed to determine the ability of AMH and INH-A to distinguish between adolescents with PCOS and controls (Figure 1): for AMH-AUC of 0.88, p<0.001, 95% CI: [0.80-0.96]; for inhibin-A -AUC of 0.74, p=0.001, 95% CI: [0.61-0.87], respectively. The cut-off value for AMH was 6.1 ng/mL, and the cut-off value for INH-A was 12.8 pg/mL to make a diagnosis of PCOS. With these cut-off values, AMH had a specificity of 92.3% and a sensitivity of 81.1% in the diagnosis of PCOS. When INH-A was used, the specificity and sensitivity were 69.2% and 86.8%, respectively. When AMH and INH-A were used in combination, the specificity and sensitivity were found as 65.4% and 96.2%, respectively (Figure 2).

## DISCUSSION

Our study is the first study in which INH-A, INH-B, and INSL-3 levels, together with AMH levels, were examined in adolescents with PCOS. In addition, we believe it is also important to note that in this study, the cut-off values with high sensitivity and specificity rates for AMH and INH-A have been determined.

WC measurement and WHR were reported to be more contributory compared with BMI in the diagnosis of metabolic syndrome (37). Visceral adiposity is a frequent finding in patients with PCOS, and this leads to hyperandrogenemia by way of insulin resistance (38,39). In our study, there was no difference between the PCOS and control groups in terms of obesity-overweight and normal weight distribution. WC SDS and WHR levels, which indicate increased visceral adiposity in adolescents with a diagnosis of PCOS, were found to be higher in the PCOS group compared with controls. Cortet-Rudelli et al (40) reported a positive correlation between FAI and the rate of visceral adiposity. A positive correlation was also shown between FAI and WC SDS in our study; AMH and INH-A were also found to correlate with WC SDS.

In the literature, results related to the diagnostic value of AMH in adolescents are conflicting. In most studies, AMH levels have been found to be increased in patients with PCOS (41,42,43). However, some publications have shown that the increase in AMH levels was not significant (44). In our study, AMH level was found to be significantly higher in patients with PCOS than in controls. In a meta-analysis study, various values were specified for the cut-off value of AMH. Sensitivity increased, but specificity decreased below 50% if this value was <3 ng/mL. When the cut-off value was accepted as >5 ng/mL, the specificity increased above 80% and the sensitivity decreased below 70% (6). In these studies which were conducted on adult women, the cut-off value was specified to be about 4.7 ng/mL. Few studies have proposed a cut-off value for AMH in adolescents. Deveer et al (45) had estimated the cut off value of AMH for adolescents with PCOS as 6.6 ng/mL. In this present study, a cut-off value of 6.1 ng/mL was shown to have a high sensitivity and specificity.

Different results have been reported in studies that examined androgens as they related with AMH in patients with PCOS. Some studies have shown that AMH closely correlated especially with testosterone (43,46), and others have shown that it correlated with D4-A and FAI (47). In our study, AMH was found to significantly correlate with androgens such as DHEAS, fT, D4-A.

INSL3 has been suggested as a laboratory test that can be used in the diagnosis of PCOS (16,48). However, these studies were conducted with adult women. In our study, adolescent girls with PCOS were compared with healthy adolescent girls, and INSL3 was not significantly different in the two groups. To date, only two studies have investigated the levels of INSL3, but these studies were conducted on healthy adolescents. Pelusi et al (17) showed that INSL3 was higher in healthy adolescents with anovulatory cycles compared with adolescents who had ovulatory cycles, but the number of subjects with anovulatory cycles was considerably limited in their study. In a study which included peripubertal healthy girls, it was proposed that INSL3 was released especially from large antral follicles (12). Accordingly, it may be thought that INSL3 has no place in the diagnosis of PCOS, but it should be noted that this study was also conducted with a limited number of subjects. In some studies, it has been reported that INSL3 was related with androgens that originate from the ovary. However, these studies were conducted on late adolescents and adults (12,15). In our study, it was also found that INSL3 positively correlated with the level of T, albeit insignificantly.

Inhibins are hormones released in the ovaries during follicular development, and their effects in patients with PCOS are still a subject of investigation. No studies have been conducted with adolescents with PCOS in this area. Studies conducted with adults have yielded controversial results. While some have shown that INH-A does not change in patients with PCOS (20,21), other studies have shown an increase (22,23,24), while some other studies reported a decrease (25,26,27). Some studies have shown that INH-B level increases in patients with PCOS (28), whereas others have shown that it decreases (30). Overall, results generally indicate that the INH-B level does not change markedly in PCOS patients (20,21,23,27,29). It has been shown that the levels of INH-A are low in the follicular phase of ovulation, increase just after ovulation, and increase further as the follicle develops (49,50). INH-B is released from small antral cells during the follicular phase (19,21). It is to be expected that the level of INH-A, which is related to increased LH level, and the level of INH-B, which is related to increased FSH level, are variable in patients with PCOS since LH and FSH levels are also variable in these patients who mostly have an increased LH/FSH ratio with LH predominance. These studies have been conducted on women with PCOS according to menstrual cycle phases, considering the periods of the menstrual cycle (21). However, patients with PCOS generally have LH predominance. Therefore, it may be expected that the level of INH-B, which is induced by FSH, is not increased and INH-A is increased together with increased LH secretion independent of the cycle in these patients who are generally oligo-/amenorrheic. Similarly, Pigny et al (23) found the level of pro-αC INH-A, which has both mature and immature forms in patients with PCOS, to be significantly increased. In the adolescents with PCOS in our study, the level of INH-A was found to be considerably increased and its sensitivity was high. In addition, the level of INH-B did not show a marked difference, a finding in accordance with many other studies (20,21,23,27,29). This finding may also be related to the fact that our patient group was especially composed of oligo-/amenorrheic adolescents. We think that studies comparing the INH-A and INH-B levels between eumenorrheic and oligo-/amenorrheic patients with PCOS may explain the conflicting results related with these two hormones.

The inverse correlation of the INH-B level with BMI has been shown in many studies, but it has also been pointed out that this correlation is not a specific finding for patients with PCOS (23,40). In our study, the patients and control groups were specifically matched in terms of BMI, and it was found that INH-B strongly inversely correlated with BMI SDS and WC SDS, while INH-A positively correlated with WC SDS. In a study conducted by Pigny et al (23), INH-A level inversely correlated with BMI, but its correlation with visceral adiposity was not mentioned. More studies are needed in this area.

In our study, INH-B was found to correlate with FSH level. This correlation may be considered an expected finding for INH-B which shows an increase with FSH secretion. However, some studies have found an inverse correlation between these two hormones (20). The correlation of INH-A with LH level and LH/FSH ratio confirmed the findings of another study (20) and regression analysis also showed the effect of LH on INH-A in our study. Correlation analysis demonstrated that INH-A also correlated with SHBG and DHEAS. Some studies have claimed that there is no correlation between INH-A and androgens in patients with PCOS, but in those studies, the comparisons were made by examining only some androgens (20,21,26). Larger scale studies are also needed in this area.

One of the limitations of our study was the fact that the ages were different in the patient and control groups. However, the fact that BMI SDS values and age of menarche were similar in the two groups, partially eliminates this limitation. Also, our control group consisted of adolescents who were eumenorrheic for at least two years. BMI-matched groups were selected to enable accurate evaluation of the hormones, because it is known that INH-B levels correlate with BMI (21,30).

In conclusion, the results of this present study indicate that the levels of INSL3 and INH-B do not have diagnostic value in adolescents with PCOS. On the other hand, it was shown that INH-A could be used as a new diagnostic biomarker in addition to AMH. Currently, we need more large-scale studies to identify biomarkers that could be helpful in adolescent cases where the diagnosis of PCOS is not definite.

## Ethics

Ethics Committee Approval: The Ethics Committee of İstanbul University Faculty of Medicine (no. 2421), Informed Consent: Written consent was obtained from all parents and participants.

Peer-review: Externally peer-reviewed.

## Figures and Tables

**Table 1:**
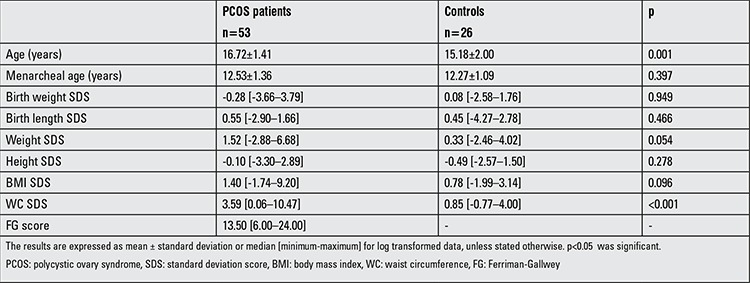
Clinical and anthropometric findings of the polycystic ovary syndrome patients and controls

**Table 2:**
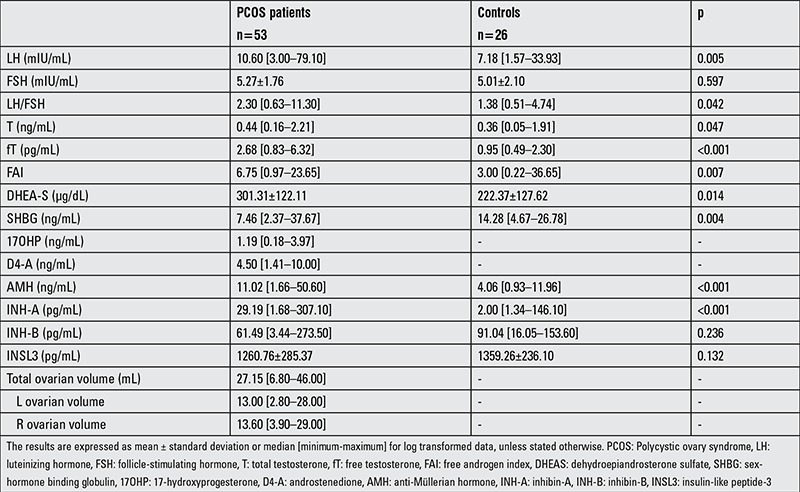
Laboratory findings in polycystic ovary syndrome patients and in the control group

**Table 3:**
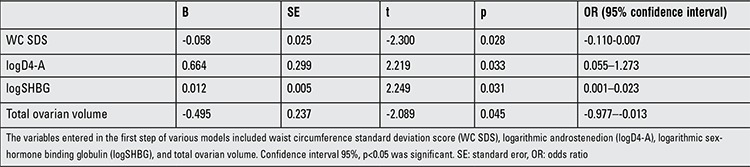
Multiple linear regression analysis of the factors associated with logarithmic anti-Müllerian hormone in polycystic ovary syndrome group (adjusted R2=0.284)

**Table 4:**
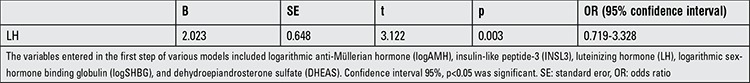
Multiple linear regression analysis of the factors associated with logarithmic inhibin-A in polycystic ovary syndrome group (adjusted R2=0.157)
